# CrMP-Sol database: classification, bioinformatic analyses and comparison of cancer-related membrane proteins and their water-soluble variant designs

**DOI:** 10.1186/s12859-023-05477-9

**Published:** 2023-09-25

**Authors:** Lina Ma, Sitao Zhang, Qi Liang, Wenting Huang, Hui Wang, Emily Pan, Ping Xu, Shuguang Zhang, Fei Tao, Jin Tang, Rui Qing

**Affiliations:** 1 State Key Laboratory of Microbial Metabolism, School of Life Sciences and Biotechnology, Shanghai Jiao Tong University, Shanghai, 200240 China; 2 Zhejiang Lab, Research Center for Intelligent Computing Platforms, Hangzhou, 311121 Zhejiang China; 3 The Lawrenceville School, 2500 Main Street, Lawrenceville, NJ 08648 USA; 4 Media Lab, Massachusetts Institute of Technology, 77 Massachusetts Avenue, Cambridge, MA 02139 USA

**Keywords:** Membrane protein, Protein design, QTY code, Machine learning, Protein function, Cancer, Bioinformatics

## Abstract

Membrane proteins are critical mediators for tumor progression and present enormous therapeutic potentials. Although gene profiling can identify their cancer-specific signatures, systematic correlations between protein functions and tumor-related mechanisms are still unclear. We present here the CrMP-Sol database (https://bio-gateway.aigene.org.cn/g/CrMP), which aims to breach the gap between the two. Machine learning was used to extract key functional descriptions for protein visualization in the 3D-space, where spatial distributions provide function-based predictive connections between proteins and cancer types. CrMP-Sol also presents QTY-enabled water-soluble designs to facilitate native membrane protein studies despite natural hydrophobicity. Five examples with varying transmembrane helices in different categories were used to demonstrate the feasibility. Native and redesigned proteins exhibited highly similar characteristics, predicted structures and binding pockets, and slightly different docking poses against known ligands, although task-specific designs are still required for proteins more susceptible to internal hydrogen bond formations. The database can accelerate therapeutic developments and biotechnological applications of cancer-related membrane proteins.

**Supplementary Information:**

The online version contains supplementary material available at 10.1186/s12859-023-05477-9.

## Background

Membrane proteins are miniscule molecular machines embedded in the phospholipid bilayer of cells that encompass essential enzymatic, signaling and molecular transporting functions in living organisms. They make up ~ 30% of genes in higher eukaryotes and account for ~ 60% of therapeutic targets for modern drugs [1]. Unsurprisingly, membrane proteins are involved in the most common forms of cancers and considered hallmarks of tumor cells. They participate in all stages of tumor progression, from initiation, invasion, growth, cellular proliferation to metastasis by mediating: (1) cell communication and signal transductions through interacting with ligands and downstream messengers [2–5]; (2) intracellular/extracellular ion homeostasis, metabolic pathways and chemoresistance [6, 7]; and (3) cell survival, proliferation and apoptosis [8]. Tumors can utilize membrane protein-regulated mechanisms to employ both the immune system and nervous system in favor of cancer progression[9–12]. Thus, great efforts are devoted to elucidate tumor possessed mechanistic pathways in specific malignancies for immunotherapy developments [13–15].

Membrane proteins’ pathological involvements are demonstrated by monitoring protein overexpression, whereas cancer-specific signatures were revealed by gene profiling [16, 17]. Correlation of their abundance with the clinical outcome of patients provides valuable insights in disease progression and prognosis [18, 19]. The research also helps to develop therapeutic strategies such as targeted drugs like monoclonal antibodies, nanocarrier drug delivery, and fluorescent tumor imaging in surgery. However, although gene patterns can reveal the significance of respective proteins in each pathology, functional studies at the molecular level are required to illuminate mechanistic processes [4].

The binding of membrane proteins with endogenous ligands and subsequent signaling are essential to explaining their functions in cancer-related biological processes [20, 21]. Mainstream ligand identification methods include radio-ligand binding, calcium flux, GTP_γ_ binding, and cAMP modulation, by exposing transcribed cells to synthetic compound libraries and observing cell activation profiles [22]. These indirect efforts are limited by the system complexity and knowledge of downstream pathways [23]. Alternative computational strategies use homologous mapping across species [24–26] or virtual screening [27] to predict interactions in different types of membrane proteins[2]. However, subsequent experimental verifications are required.

The major obstacle against structure determination, ligand identification and mechanism studies of membrane proteins is their hydrophobicity and tendency to aggregate in aqueous solutions [28, 29]. Common stabilization methods such as detergent screening or nanodiscs require arduous individual efforts, and are difficult to push beyond research purposes [30]. The advent of AlphaFold2 partially resolved this issue, which is a computational tool for protein structure predictions [31, 32]. The deep-learning architecture uses co-evolution information and homologous crystal structures in the Protein Data Bank (PDB) to conduct accurate simulations. The program and its predicted structures for nearly all catalogued proteins with sequence information known to science are publicly available [33, 34].

Another experimental approach to circumvent such issues is through a rational design tool we previously devised that named QTY code [35]. The water-soluble and functionally equivalent variants of native membrane proteins can be easily designed through pairwise amino acid substitutions [35, 36]. Specifically, hydrophobic residues of Leucine (L), Valine (V) and Isoleucine (I), and Phenylalanine (F) in the transmembrane (TM) region are substituted by hydrophilic Glutamine (Q), Threonine (T), and Tyrosine (Y), respectively. The methodology was demonstrated first on chemokine receptors [35], and later used to elucidate structural basis of their ligand recognitions and regulatory role in vivo [35, 36]. Additional bioinformatic studies were conducted which applied this protocol on different classes of membrane proteins [32, 37, 38]. It is proposed that these detergent-free membrane proteins can be adopted to conduct screening in solution for ligand identification from a biophysiochemical aspect.

To date, despite extensive efforts to establish a membrane protein mediated network of human cancers [2, 4, 39], there is not yet a database to provide essential reference information for cancer-related researches with respect to the understanding of protein functions and molecular mechanisms. The systematic correlation between membrane proteins and tumor pathogenesis are still lacking beyond their cancer-specific signatures revealed by gene profiling. Here we present CrMP-Sol (Cancer-related Membrane Protein and Solubilization database), which is dedicated to connecting molecular characteristics and biological functions of membrane proteins to their participation in cancer pathology, while presenting water-soluble designs to facilitate native membrane protein research.

The database contains 1309 entries related to 17 types of cancers, which were classified into 7 categories, and plotted into 3D-space using machine learning algorithms based on extraction of key functional descriptions. The spatial distribution can be used to predict inapparent relations between adjacent proteins and specific pathogenesis through common mechanisms beyond genetic level analysis. The QTY code was employed for water-soluble designs to facilitate native membrane protein studies in spite of natural hydrophobicity on all 1309 proteins in the database. Five exemplary proteins from different categories and varying numbers of TM helices were used for feasibility demonstration. The QTY variants exhibited highly similar characteristics and structurally superimposed well with native proteins, in addition to enhanced hydrophilicity and stability. Beyond the scope of prior works, we performed comparative analysis on molecular dockings of native and QTY variant proteins against native ligands that might be involved in different pathogeneses. The docking showed slightly altered poses and closely-matched binding energies. Channel-forming proteins exhibited best agreements in geometry and hydrogen bonding sites. For binding pairs with significant changes in conformations and binding energies, molecular dynamic (MD) simulations revealed the decreased hydrophobic interactions to be accountable for the differences.

Our database provides essential information to connect and predict correlation between membrane protein functions and cancer types. The unraveling of hidden relations encoded within biomolecular processes and mechanistic pathways in specific malignancies can shed light on new research directions not apparent from gene-level analysis. The water-soluble designs are also presented in our database as an experimentally feasible solution to facilitate subsequent researches, by offering physical simulators of native membrane proteins. Verification and regulation of these potentially indispensable biological processes can not only provide new scientific insights on the initiation and progression of diseases, but also benefit corresponding therapeutic developments and other biotechnological applications.

## Results

### CrMP-Sol database

Information of cancer-related membrane proteins at the genetic level are based on a previous transcriptome study, which is available on *The Human Protein Atlas* (HPA, https://www.proteinatlas.org/) [40–42]. Out of 20,090 entries in the database, 11,279 of the proteins are associated with cell membranes [43], where 1309 proteins are clinically relevant to 17 types of cancers, including: colorectal cancer, endometrial cancer, melanoma, renal cancer, liver cancer, testis cancer, pancreatic cancer, glioma, thyroid cancer, prostate cancer, cervical cancer, lung cancer, urothelial cancer, breast cancer, head and neck cancer, stomach cancer, and ovarian cancer [41]. We classified these entries into 7 categories based on descriptions of their functions, which included 327 receptors, 161 transporters, 44 carriers, 124 channels, 201 enzymes, 109 contact proteins, and 344 others lacking apparent functional classifications. Other information about gene and protein expressions, distributions in organs, cell lines, immune cells and bloods are also available in the database [43].

Besides pathogenesis data, critical genetic and molecular information regarding the protein functions are also presented in CrMP-Sol, which referred to NCBI (National Center for Biotechnology Information), Uniprot and PDB. Genetic information consists of gene name, location, a summary of the gene encoding the protein, and open-source links. Molecular information includes name, primary sequence, subcellular locations, crystal and AlphaFold2 predicted structures, and descriptions about experimentally verified or proposed protein functions. The tissue and pathogenesis specificity are also presented.

As a core feature of our database, we designed water-soluble variants of all 1309 membrane proteins by QTY code [44]. Specifically, the primary sequences of these QTY variants, AlphaFold2 predicted structures, and superimpositions with native proteins are presented. It is proposed that these easy-to-synthesize, cost-efficient, more hydrophilic structural and functional equivalents of naturally hydrophobic proteins can accelerate molecular and mechanistic study of the latter to facilitate the development of cancer treatments. These novel water-soluble variants of membrane proteins may also themselves be adopted in therapeutic applications [45].

### Classification and visualization of protein-cancer types

To intuitively establish correlation between protein functions and cancer specificities, we encoded data entries with functional descriptions and visualize them in a 3D-space. The TF-IDF (frequency-inverse document frequency) machine-learning algorithm was adopted to extract keywords based on their relative frequency of appearances in each description compared to the whole database, to distinguish minor functional differences in proteins [46]. Words not directly related to protein functions like PubMed ID were manually removed. As the most important hyperparameter for TF-IDF, the number for max features (MF) was adjustable in the interface with cut-offs between 50 and 250 words and a step size of 50. This step allows users to choose either the most important or more inclusive descriptions of protein functions for tailored classifications, without making the data matrix non-efficiently large.

**Fig. 1:**
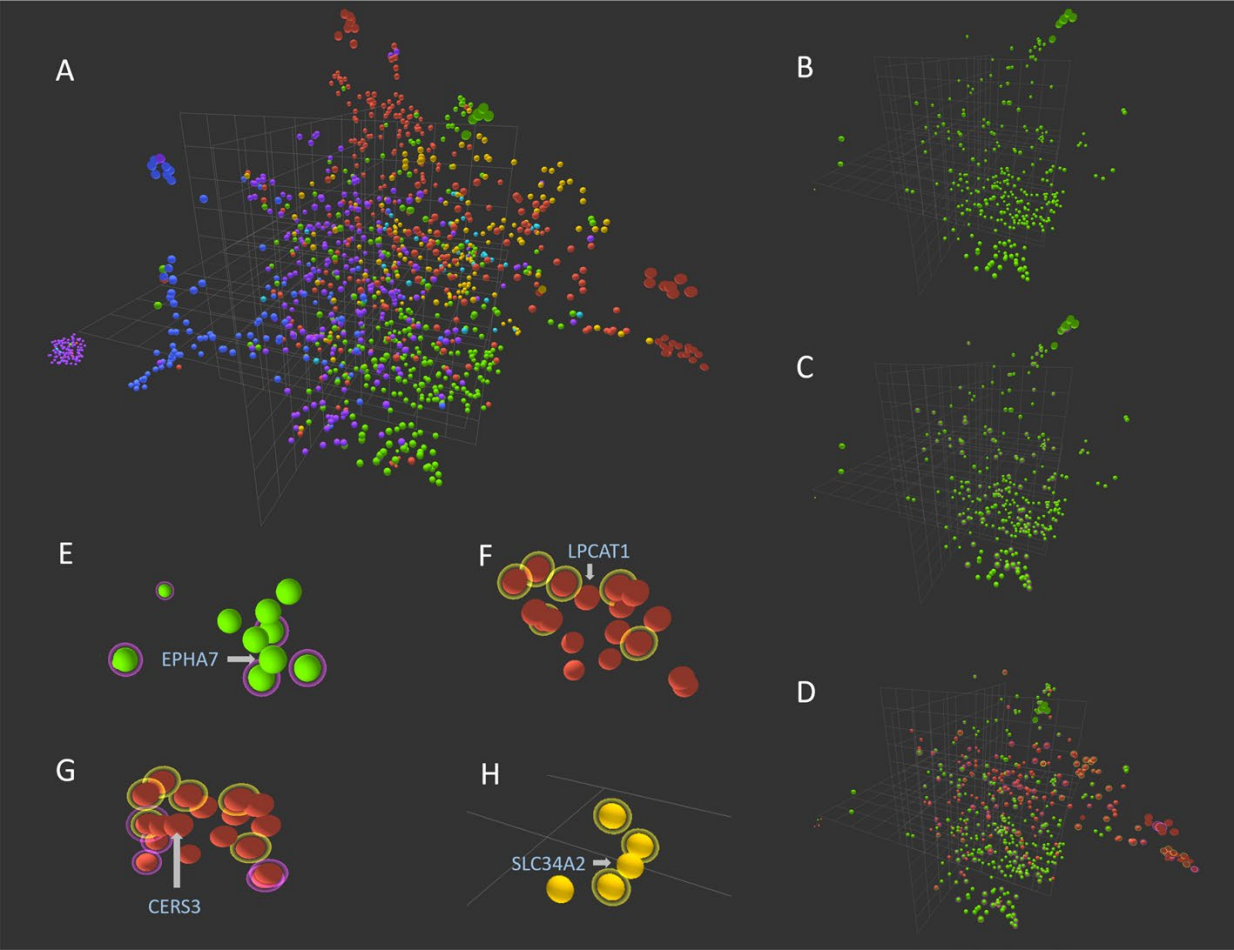
Spatial distribution of cancer-related membrane proteins in 3D-space based on TF-IDF analysis on their functional descriptions with UMAP dimension reduction algorithm. **A** The interactive interface with all proteins shown. **B** The interface showing only receptor proteins. **C** The interface highlighting receptor proteins related to glioma cancer. **D** The interface highlighting receptor and enzyme proteins related to glioma and liver cancer. **E** The EPHA7 datapoint resides in close proximity of receptors associated with glioma (purple halo). **F** The LPCAT1 datapoint resides closely to enzymes that are associated with liver cancer (yellow halo). **G** The CERS3 datapoint resides in a pocket formed by proteins associated with glioma (purple halo), liver cancer (yellow halo), or both (dual halo). **H** The SLC34A2 data point resides near transporters that are associated with liver cancer (yellow halo)

A 1309 × MF matrix was then established to represent the protein × function information. The UMAP (Uniform Manifold Approximation and Projection for Dimension Reduction) algorithm was adopted to reduce the dimension of encoded data while preserving its global structure and visualizing in a 3D-coordinate system (Fig. 1A). In this low-dimensional space, protein classifications were denoted by different colors, while halos around a single datapoint represented cancer types. The distant purple cluster at top-left corner represents entries currently without functional descriptions. The interactive graph is the front page of our database, where users can select a single datapoint to access the detailed information page. The interface also allows the selection and highlighting of each protein category, or those associated with one or several types of cancers (Fig. 1B–D). The feature provides information of membrane proteins or critical mechanistic processes adopted by different pathologies in each category.

Beyond the apparent information that the same types of proteins exhibit relative clustering in the 3D-space, we hypothesize that the graph also reveals functional connections encoded by dimension reduction. It is likely that adjacently positioned proteins have higher chance to participate in functionally relevant pathways contributing to the same pathology, whether or not they exhibit concurrent profiling in the gene analysis. For instance, when “receptor” and “glioma” were selected, we found datapoint EPHA7 (Ephrin type-A receptor-7) not overexpressed in the gene-level, but was in close proximity of several receptors all associated with the cancer (Fig. 1E). Literature review indeed suggested its relation to malignant glioma despite genetic analysis labeling it as irrelevant [47]. Similarly, LPCAT1 is adjacent to five enzymes related to liver cancer. Its expression was found to enhance the phosphatidylcholine level in hepatocellular carcinoma tissues, which promoted cellular proliferation, migration, and invasion [48]. On the other hand, CERS3 (Ceramide synthase 3) resides in a wide pocket of 9 proteins related to glioma, liver cancer, or both (Fig. 1G). Despite its normal transcription level in either pathology, a recent study found the enzyme to affect invasion and metastasis of hepatocellular carcinoma via SMAD6 gene [49], whereas it also regulates AKT/ERK1/2 signaling critical for angiogenesis of glioblastoma [50]. Furthermore, as shown in Fig. 1H, there are three other liver cancer-related transporters adjacent to SLC34A2 (Solute carrier family 34 member), while the knockdown of the latter was also found to inhibit hepatocellular carcinoma cell proliferation and invasion [51]. The overall reliability of prediction efficacy will need more extensive evaluation based on data mining and preferably dedicated experimental validation. Yet the few examples presented here already showed the prospect of integrating functional information beyond genetic-level analysis into the clusters of proteins with correlation to pathologies.

### QTY design and property comparisons

The design of water-soluble variants likely provides mechanistic insights for native membrane proteins and accelerate therapeutic developments, as has been demonstrated before [36, 52]. Thus, we conducted QTY design on all 1309 cancer-related membrane proteins in the database. The L, I, V, F residues in the TM region of native proteins were replaced by Q, T and Y accordingly in the designs (with T replacing both I and V). The process was conducted using an automated online PSS server established prior [44].

Since we cannot present all designed sequences in one paper, five proteins of different categories with varying numbers of TM helices are selected as exemplary demonstrations, including MGAT3 (Monoacylglycerol O-Acyltransferase 3), GPR35 (G protein-coupled receptor 35), GPR37 (G protein-coupled receptor 37), SLC10A1 (Solute carrier family 10 member 1), and NPC1L1 (Hepatic Niemann-pick C1-like 1). MGAT3 is a 3TM enzyme commonly expressed in the gastrointestinal tract that catalyzes the synthesis of 1,2-diacylglycerol from 2-monoacylglycerol and has a role in dietary fat absorption [53]. It is relevant to colorectal cancer, liver cancer and stomach cancer. Both GPR35 and GPR37 belong to the G-protein coupled receptor family with 7TM helices. They regulate osteogenesis via the Wnt/GSK3β/β-catenin pathway [54], or bind prosaptide to enhance ERK signaling and inhibit cAMP levels [55]. GPR35 is related to colorectal cancer, pancreatic cancer and stomach cancer, while GPR37 is related to glioma, melanoma and liver cancer. SLC10A1 is a 8TM solute carrier co-transporter primarily localized in hepatocytes, and plays a key role in bile acid extraction and biliary excretion from portal blood [56]. The protein hosts hepatitis B virus infection and is associated with liver cancer [57]. NPC1L1 is a large 13TM polytopic sterol transporter localized at the apical membrane of enterocytes and the canalicular membrane of hepatocytes [58]. It serves as a critical mediator for cellular cholesterol uptake and is involved in liver cancer, pancreatic cancer and stomach cancer [59].

**Fig. 2:**
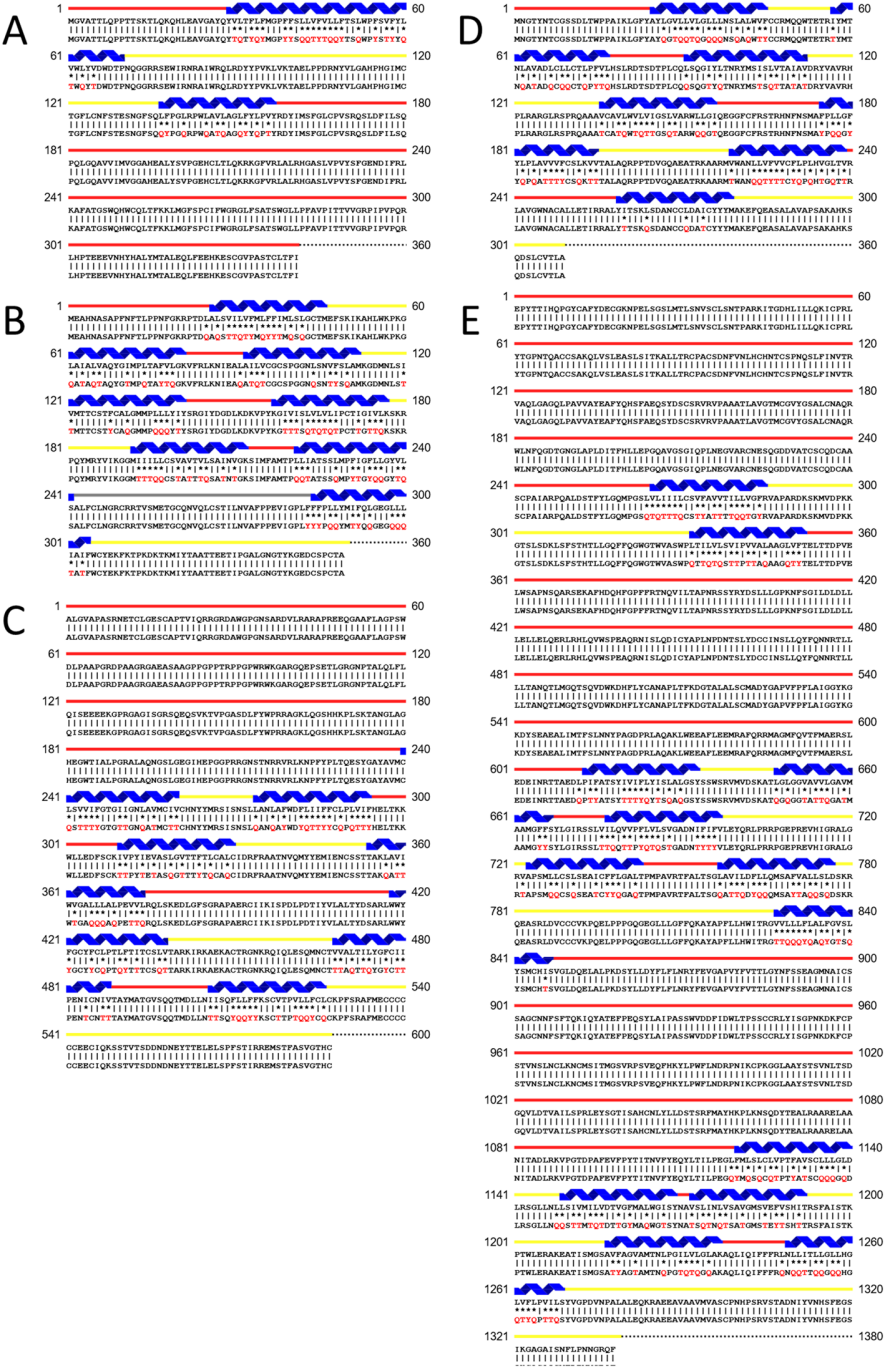
Sequence alignments of 5 cancer-related membrane proteins with their water-soluble QTY variants. The alignments are: **A** MGAT3 versus MGAT3^QTY^, **B** GPR35 versus GPR35^QTY^, **C** GPR37 versus GPR37^QTY^, **D** SLC10A1 versus SLC10A1^QTY^, and **E** NPC1L1 versus NPC1L1^QTY^. The Q, T, and Y amino acid substitutions are in red. The α-helical segments (blue) are shown above the protein sequences, the external (red) and internal (yellow) loops of the receptors are indicated. The symbols | and * indicate the unchanged and changed amino acids, respectively

Sequence alignments of QTY designed water-soluble proteins and their native counterparts are shown in Fig. 2. Individual optimizations were not conducted for this mass-design process. QTY substitutions were applied to all corresponding residues only in the TM region, but not those in extracellular domains and intracellular domains.

The protein characteristics were calculated and compared in Table 1. Despite significant QTY substitutions on LIVF residues in TM regions (~ 48–54%), the isoelectric point (pI) and molecular weight (MW) of QTY proteins are quite similar to native proteins. This is due to that, although Q, T and Y can induce the formation of intra-, inter- and solvent-exposed hydrogen bonds, they do not carry additional charges. The substitutions enhance the protein solubility while retaining its overall integrity without introducing additional disruptive electrostatic interactions. The alteration of hydrophobicity in the helical region of membrane proteins without changes in steric and electrostatic interactions is the essence of QTY code. The slight MW increase is due to the introduction of hydroxyl group in respective residues.

**Table 1:** Characteristics of native membrane proteins and their water-soluble QTY variants

Name	pI	MW (kDa)	TM variation (%)	Total variation (%)	RMSD (Å)
MGAT3	8.86	38.73	–	–	0.157, 0.309 (TM)
MGAT3^QTY^	8.79	39.13	52.38	9.68	
GPR35	9.06	34.07	–	–	1.478, 1.044 (TM)
GPR35^QTY^	9.01	34.65	49.32	23.62	
GPR37	8.43	64.35	–	–	1.216, 0.899 (TM)
GPR37^QTY^	8.41	64.79	52.38	13.12	
SLC10A1	9.07	38.11	–	–	1.233, 0.544 (TM)
SLC10A1^QTY^	8.99	38.64	48.81	24.64	
NPC1L1	5.90	146.37	–	–	0.656, 0.603 (TM)
NPC1L1^QTY^	5.90	147.50	53.48	11.43	

Isoelectric focusing (pI), Molecular weight (MW), Transmembrane (TM), – = not applicable. The internal and external loops have no changes, the overall changes are not insignificant, and the TM changes are large

### Superimpositions of AlphaFold2 predicted structures of native and QTY cancer-related membrane proteins

**Fig. 3:**
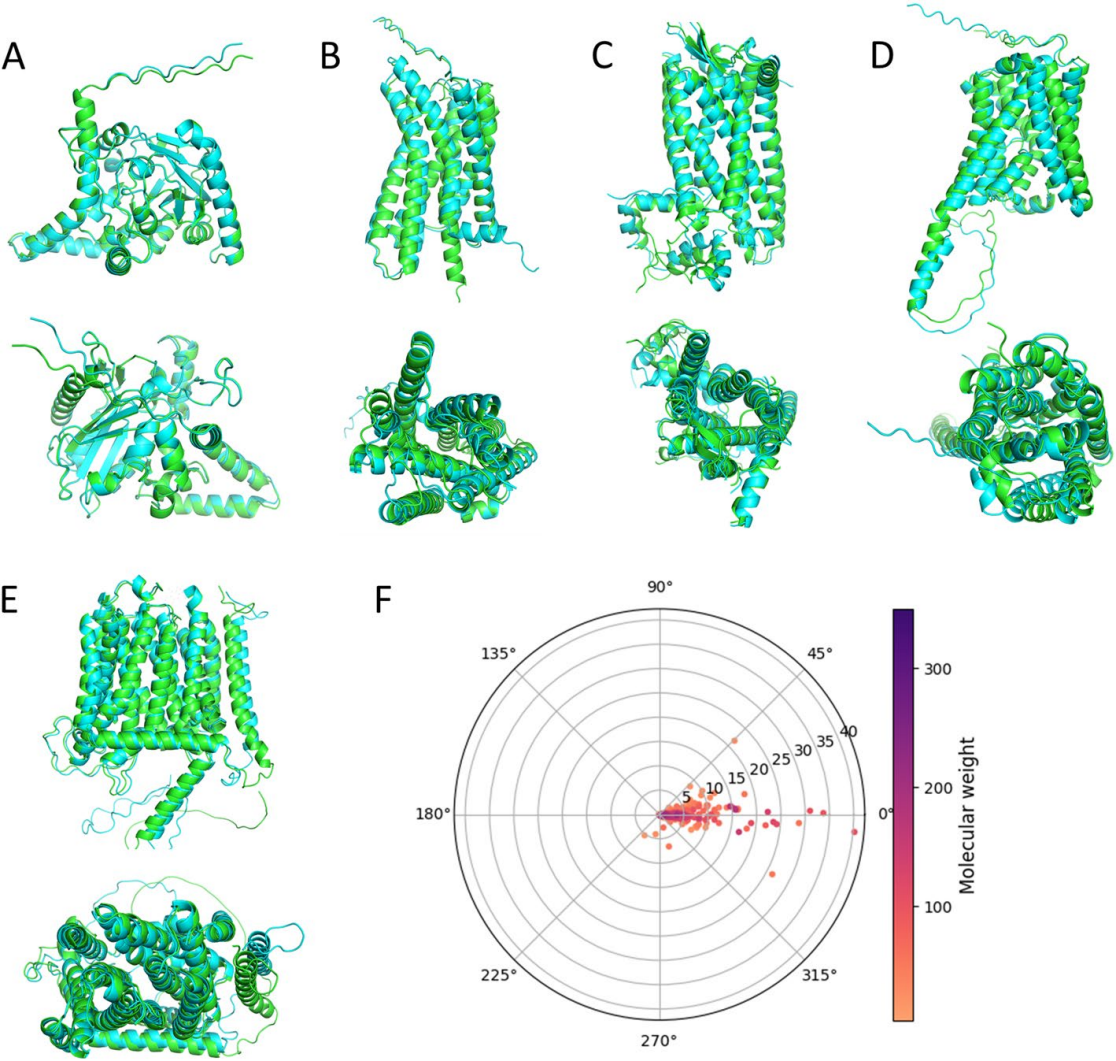
Superposed AlphaFold2 predicted 5 cancer-related membrane proteins (green) with their water-soluble QTY variants (cyan). Side view and top view are presented. For clarity, both extracellular and intracellular regions are removed. **A** MGAT3 versus MGAT3^QTY^ (RMSD: 0.157, 0.309 (TM)), **B** GPR35 versus GPR35^QTY^ (RMSD: 1.478, 1.044 (TM)), **C** GPR37 versus GPR37^QTY^ (RMSD: 1.216, 0.899 (TM)), **D** SLC10A1 versus SLC10A1^QTY^ (RMSD: 1.233, 0.544 (TM)), and **E** NPC1L1 versus NPC1L1^QTY^ (RMSD: 0.656, 0.603 (TM)). **F** RMSD distribution of the 1309 QTY designs for all cancer-related membrane proteins in the database. Each dot represents a QTY design, with the RMSD value corresponding to the distance from the origin. The angle in the polar coordinate system represents the degree of the secondary structure change. Higher angles in respect to the horizontal line represents greater secondary structure change. A color gradient represents the molecular weight of each protein

The structural similarity between QTY designed MGAT3, GPR35, GPR37, SLC10A1, NPC1L1, and native counterparts were demonstrated by comparing AlphaFold2 predicted structures. The predicted structures were validated by ProSA web tool and reported as z-score values [60]. Lower z-scores correspond to higher model validity, where predicted structures of native and QTY variant generally exhibited closely matched z-score values (Additional file 1: Table S1). As shown in Fig. 3, predicted structures for native and QTY proteins superimposed very well. Both side views and top views of the superimpositions are shown. Despite > 48% changes in TM sequences, the RMSD (root mean square deviation) for two protein variants under investigation are < 1.5 Å, suggesting very high conformational similarities. Specifically, RMSDs for MGAT3 versus MGAT3^QTY^, GPR35 versus GPR35^QTY^, GPR37 versus GPR37^QTY^, SLC10A1 versus SLC10A1^QTY^, and NPC1L1 versus NPC1L1^QTY^ are 0.157 Å, 1.478 Å, 1.216 Å, 1.233 Å, and 0.656 Å, respectively. TM region RMSDs for MGAT3 versus MGAT3^QTY^, GPR35 versus GPR35^QTY^, GPR37 versus GPR37^QTY^, SLC10A1 versus SLC10A1^QTY^, and NPC1L1 versus NPC1L1^QTY^ are 0.309 Å, 1.044 Å, 0.899 Å, 0.544 Å, and 0.603 Å, respectively. Improvements on TM region RMSDs were attributed to the deletion of intrinsically flexible loop domains that contribute more to the RMSDs, which further demonstrated the applicability of QTY methodology on TM helices without structural alterations [61, 62].

Despite that we cannot show superimpositions of all 1309 membrane proteins in this article, the RMSDs between native and QTY variants, along with MW and secondary structure changes were summarized and plotted in Fig. 3F. Most redesigned proteins exhibit RMSD values < 10 Å, with the densest distribution below 5 Å. The outliers are relatively darker in color, suggesting their higher MWs and more complex structures. Moreover, there are only a few designs falling outside the ± 45° sectors in the graph, while most datapoints reside close to the horizontal line. This suggests that most native and QTY variant proteins share similar secondary structures.

### Hydrophobicity analysis of native and QTY cancer-related membrane proteins

**Fig. 4:**
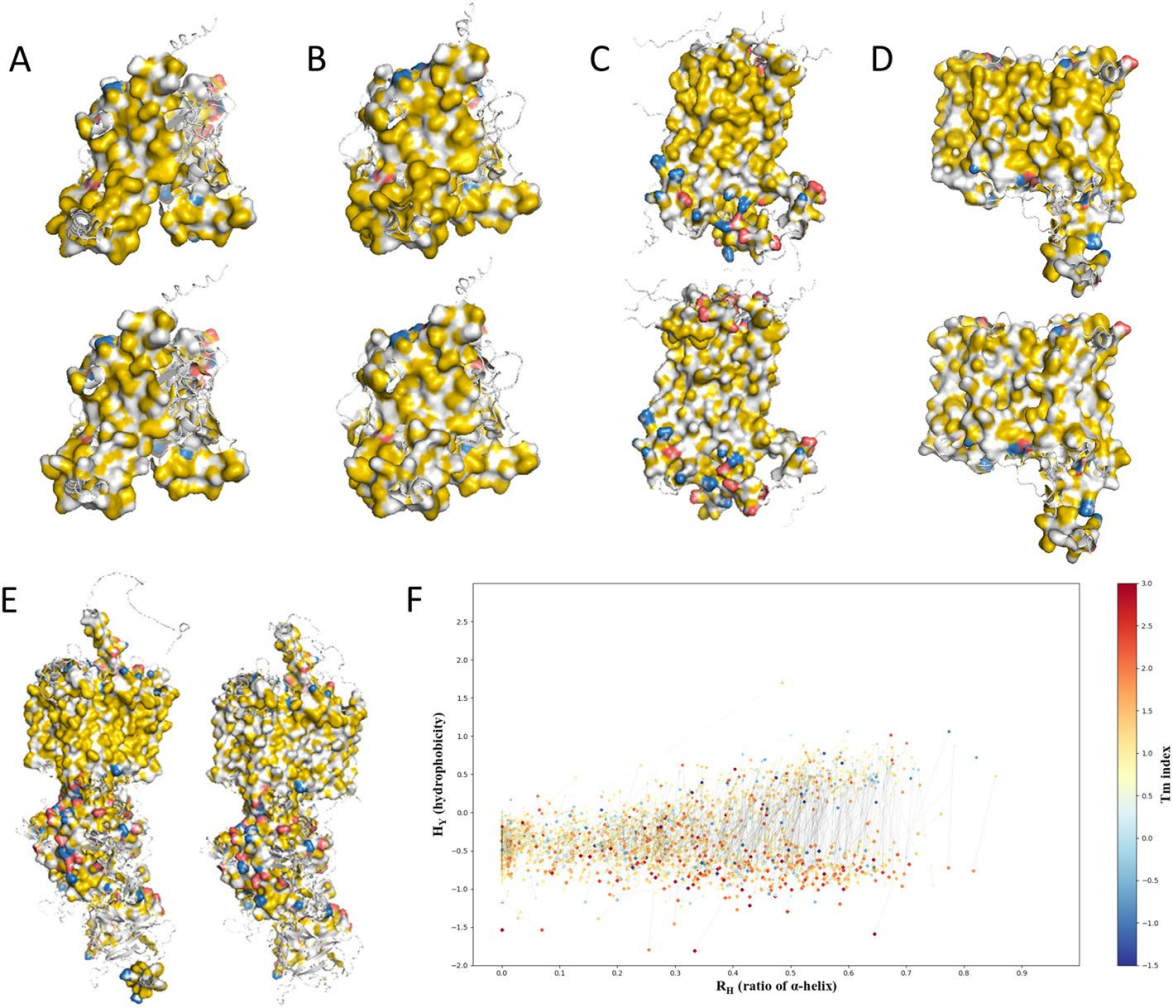
The pairwise hydrophobic surface patch (brown) predictions of 5 cancer-related membrane proteins with their water-soluble QTY variants. For clarity, both extracellular and intracellular regions are removed. The native proteins are on top (**A–D**). **A** MGAT3 (top) versus MGAT3^QTY^ (bottom), **B** GPR35 (top) versus GPR35^QTY^ (bottom), **C** GPR37 (top) versus GPR37^QTY^ (bottom), **D** SLC10A1 (top) versus SLC10A1^QTY^ (bottom), and **E** NPC1L1 (left) versus NPC1L1^QTY^ (right). **F** Global R_H_–H_Ƴ_ distribution of the 1309 QTY designs for all cancer-related membrane proteins in the database. The R_H_ indicates the content of α-helices in a protein. The hydrophobicity (H_Ƴ_) was calculated using the ProPAS and used for evaluating the water solubility of a protein. The T_m_ index shown in color gradient was calculated using a sequence-based method, which qualitatively represents the stability of a protein. The original membrane proteins are denoted by circles, and the QTY-designed variants are denoted by diamonds. The thin black line shows the corresponding relationship between the original protein and its variant

To computationally evaluate the solubilization efficacy of cancer-related membrane proteins, we conducted bioinformatic simulations on surface hydrophobic patches of both native and QTY variant proteins. Due to the proteins being naturally embedded in the phospholipid bilayer, native proteins were surrounded by nonpolar residues at the exterior of TM helices, which represents the majority of water-repelling surfaces as colored yellow in Fig. 4A–E (top). After the QTY code was applied, the hydrophobic patches (bottom) have notably decreased compared to their native counterparts, indicating an enhanced capability for water molecule interactions in the QTY variants.

A distribution map containing hydrophobicity information of all 1309 membrane proteins was shown in Fig. 4F. R_H_ corresponds to the ratio of α-helical content in the protein, while H_Ƴ_ represents calculated hydrophobicity using ProPAS. As expected, more significant decreases in hydrophobicity are observed for proteins with higher TM helical contents, which were the targets for the QTY design with amino acid substitutions. On the other hand, by comparing the color distribution of circles (native proteins) and diamonds (QTY proteins), slight increases of T_m_ (melting temperature) were predicted for solubilized proteins using a sequence-based method, indicating relatively higher protein stability [63]. Though accurate T_m_ values will require experimental determinations, the predicted trend agrees with previous experimental findings [36]. Since water-solubility and structural stability are interconnected characteristics, it is possible that by designing more soluble proteins, we also provide a plausible method for their stabilization, which has both theoretical and practical significances [64].

### Molecular docking of native and water-soluble cancer-related membrane proteins

Preliminary functional comparison of native and water-soluble variants of cancer-related membrane proteins was conducted by docking their known ligands into predicted binding sites. The examination of computed binding geometries contributed to the understanding of molecular interactions from both conformational and compositional aspects [65]. We continued using the five exemplary proteins as in previous tasks. Both small molecule ligands and protein binders were checked. Specifically, we conducted molecular dockings for the following binding pairs: MGAT3 versus DAG (diacylglycerol), 2-MAG (2-monoacylglycerol) and oleoyl-CoA; GPR35 versus cGMP, kynurenic acid, lysophosphatidic acid, pamoic acid and Zaprinast; GPR37 versus neuroprotection D1, Osteocalcin and Saposin C; SLC10A1 versus bile acid, estrone sulfate, GCDC (glyco-chenodeoxycholic acid) and taurosholate; NPC1L1 versus cholesterol. Amongst the listed ligands, Osteocalcin and Saposin C are protein binders, whilst all others are small molecule ligands.

**Fig. 5:**
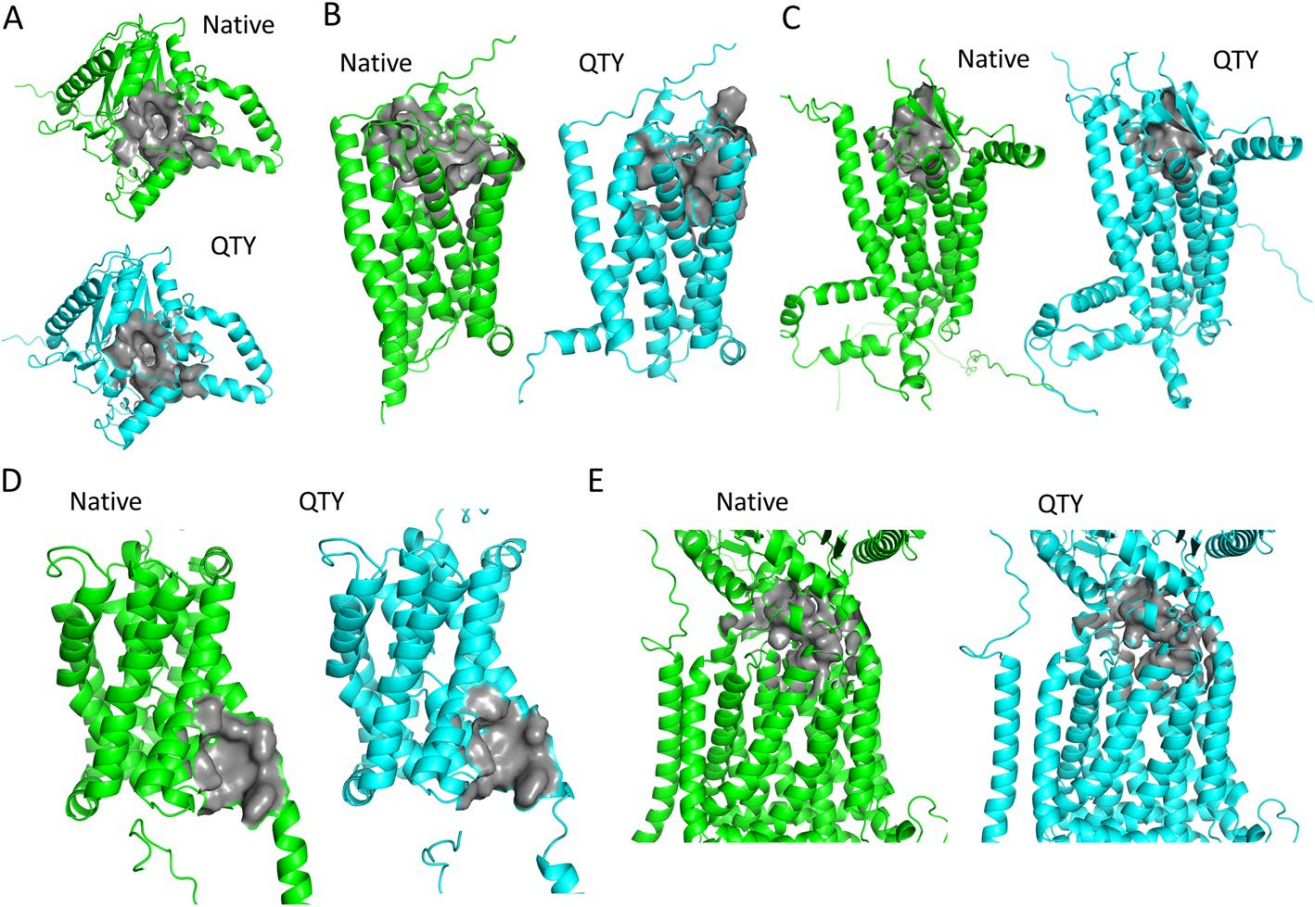
PrankWeb predicted binding pockets (gray) of 5 cancer-related membrane proteins (green) with their water-soluble QTY variants (cyan). **A** MGAT3 (top) versus MGAT3^QTY^ (bottom), **B** GPR35 (left) versus GPR35^QTY^ (right), **C** GPR37 (left) versus GPR37^QTY^ (right), **D** SLC10A1 (left) versus SLC10A1^QTY^ (right), and **E** NPC1L1 (left) versus NPC1L1^QTY^ (right). The gray color areas are the predicted binding packets

The binding pockets were predicted by PrankWeb for both native and QTY variant proteins [66]. Rational considerations were used to select a model from top 3 predictions. For MGAT3, GPR35 and SLC10A1, the highest scoring pockets were selected for subsequent docking. Yet for GPR37, the pocket 1 and 2 of native and pocket 1 of QTY protein were predicted at the C-terminus, thus pocket 3 for native protein and pocket 2 for QTY protein residing on the N-terminus were used for docking. NPC1L1 mediates cholesterol uptake by transporting it across the membrane, which involves the interaction of cholesterol with TM channels. While the 4 highest scoring pockets all resided in the extracellular region far from the phospholipid membrane and were most likely relevant to interaction with cholesterol, we intentionally selected pocket 3 for both native and QTY variants near the N-terminal entrance of the TM channel to elucidate the impact of the QTY design on the cross-lipid transportation. As shown in Fig. 5, predicted binding pockets generally agreed well between native and QTY variant proteins, providing basis for similar binding interactions.

**Fig. 6:**
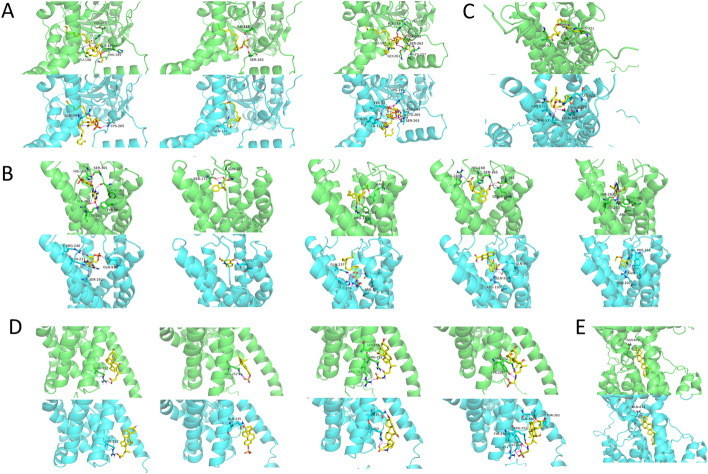
Molecular docking comparisons of 5 cancer-related membrane proteins (green, top) with their water-soluble QTY variants (cyan, bottom) against native ligands. **A** From left to right: MGAT3 versus DAG, MGAT3 versus 2-MAG, MGAT3 versus oleoyl-CoA; **B** from left to right: GPR35 versus cGMP, GPR35 versus kynurenic acid, GPR35 versus lysophosphatidic acid, GPR35 versus pamoic acid, GPR35 versus Zaprinast; **C** GPR37 versus neuroprotection D1; **D** from left to right: SLC10A1 versus bile acid, SLC10A1 versus estrone sulfate, SLC10A1 versus GCDC, SLC10A1 versus taurosholate; **E** NPC1L1 versus cholesterol

Dockings between protein models and respective ligands were performed using AudoDock Vina [67]. Simulations for each protein–ligand pair were repeated at least three time to generate a reliable docking conformation and statistically meaningful binding energies. As shown in Fig. 6A–E, despite significant amino acid changes in TM regions, the binding between proteins and their respective ligands on the QTY variants generally occurred at closely-matching locations on the native protein. However, slight docking conformation differences were observed due to the inevitable changes to local environments, with some hydrogen bonds altered at new sites. These alterations can be attributed to interference from increased numbers of polar residues, which previously did not exist in the TM helices. Extensive internal hydrogen bond networks in QTY proteins may also lead to significant changes in ligand binding poses, as shown in MGAT3:2-MAG, MGAT3:oleoyl-CoA, and GPR35:pamoic acid. The orientations of the ligands were inverted, as previously outward-facing hydrophilic segments of the molecules were drawn by the polar core of QTY proteins, leaving hydrophobic segments to face solvents uncompensated. Such changes might not only impose additional energy penalties in docking, but also possibly negate the function associated with the binding events, such as the catalytic function in MGAT3. On the other hand, the channel forming proteins, namely SLC10A1 and NPC1L1, exhibited higher agreements both on the ligand docking poses and interaction sites between the native and QTY variants, with the best-performing pair being NPC1L1:cholesterol. Almost identical poses and identical hydrogen bond formations were observed. It was deduced that the presence of high aspect ratio TM channels was likely to guide the binding and orientation of respective ligands. The transporting function was also most likely retained despite significant changes in amino acid sequences.

Table 2 summarizes the calculated binding energy (kcal/mol) for each protein–ligand pair extrapolated from AutoDock Vina. In general, QTY variant proteins showed slightly decreased binding energies as compared to their native counterparts, but were still close in numbers. The trends agreed well with our previous experimental results that QTY proteins generally exhibited very slightly lower binding affinities compared to native proteins [35, 36, 45]. It was also supported by docking pose observations, where both native and QTY variants bound to respective ligands in similar manners, despite the more complex internal hydrogen bond networks of the latter being slightly unfavorable towards intermolecular interactions. Amongst all, the GPR35:pamoic acid pair exhibited the largest binding energy discrepancy of 2.0 kcal/mol. An alternative route was conducted to evaluate this binding pair, where AlphaFold_multimer was employed to predict GPR35/G_α_ complex structure and established a model for subsequent docking (Additional file 1: Fig. S1) [68]. Almost identical docking positions and orientations were observed for the complex model (Additional file 1: Fig. S2) and those presented in Fig. 6B. Additional MD simulations on this binding pair will be presented in a later section. However, it should be noted that most of our docking computations did not consider the states of membrane proteins, complex with downstream biomolecules such as G-proteins, and potential small molecule induced conformational changes. This might render the simulated structures and calculated binding energies to have slight deviations when compared to the actual binding states of ligands, which should be determined in subsequent crystallographic studies.

**Table 2:** Binding energies for ligands versus native membrane proteins and their water-soluble QTY variants

Protein name	Ligand	Binding energy (kcal/mol)	
		Native	Water-soluble
MGAT3	Diacylglycerol (DAG)	− 6.8 ± 0.5	− 6.6 ± 0.3
	2-Monoacylglycerol (2-MAG)	− 6.6 ± 0.5	− 6.1 ± 0.2
	Oleoyl-CoA	− 7.9 ± 0.3	− 7.8 ± 0.2
GPR35	cGMP	− 8.1 ± 0.0	− 7.6 ± 0.0
	Kynurenic acid	− 6.6 ± 0.0	− 6.5 ± 0.0
	Lysophosphatidic acid	− 6.5 ± 0.2	− 6.0 ± 0.1
	Pamoic acid	− 9.9 ± 0.0	− 7.9 ± 0.1
	Zaprinast	− 7.5 ± 0.1	− 6.8 ± 0.1
GPR37	Neuroprotection D1	− 6.3 ± 0.3	− 5.9 ± 0.2
SLC10A1	Bile acid	− 8.0 ± 0.0	− 6.8 ± 0.2
	Estrone sulfate	− 8.3 ± 0.0	− 7.7 ± 0.0
	Glyco-chenodeoxycholic acid (GCDC)	− 7.7 ± 0.2	− 7.9 ± 0.1
	Taurocholate	− 7.9 ± 0.0	− 7.4 ± 0.1
NPC1L1	Cholesterol	− 7.3 ± 0.1	− 6.8 ± 0.3

**Fig. 7:**
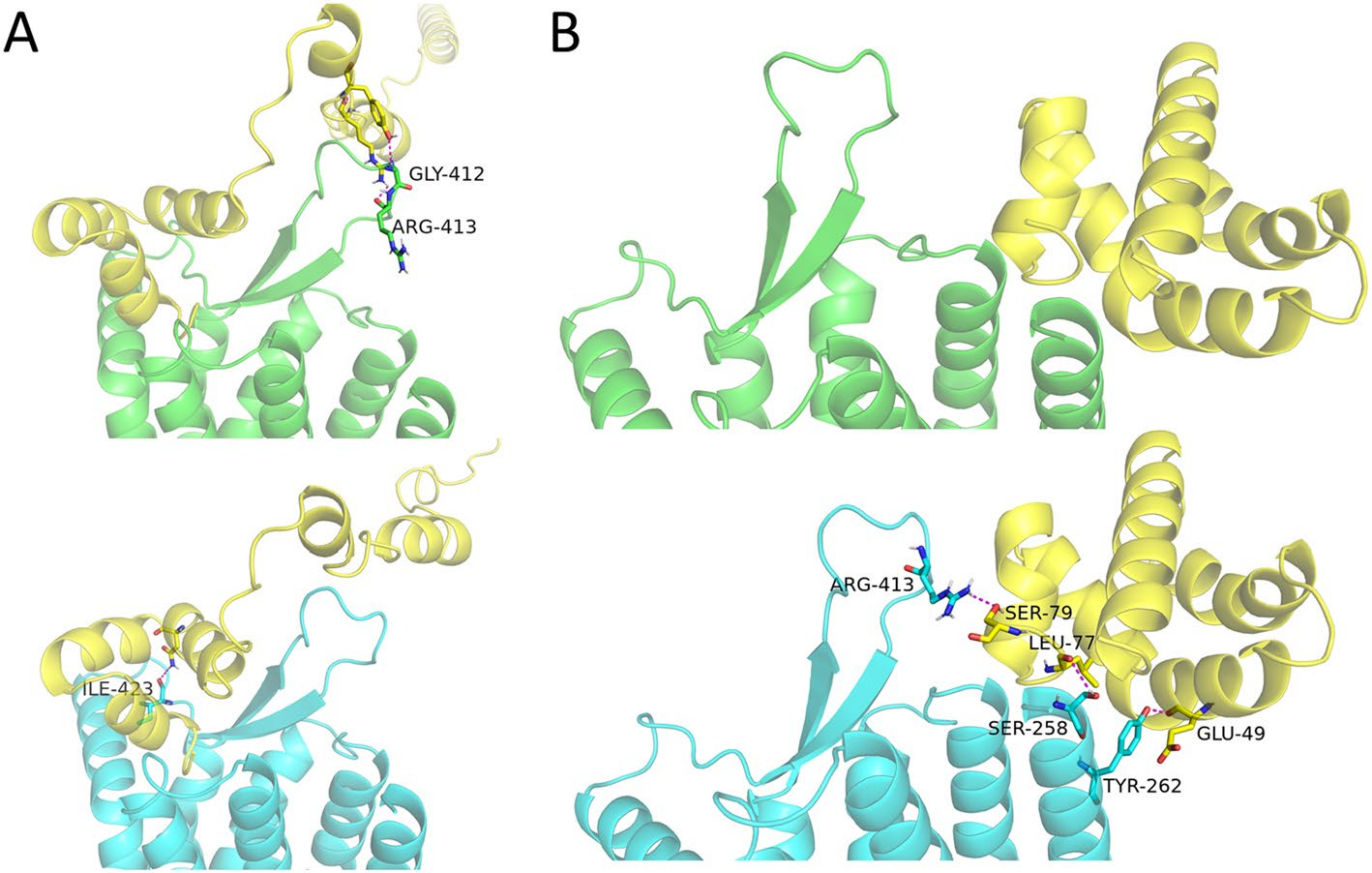
Molecular docking comparisons of GPR37 (green, top) with GPR37^QTY^ (cyan, bottom) against protein binders (yellow): **A** Osteocalcin, **B** Saposin C

Beside small molecule ligands, protein binders also play critical roles in the function of membrane proteins [61, 62]. We here used ZDOCK software to inspect the interactions of GPR37 versus Osteocalcin and Saposin C. The TM and intracellular regions were blocked for binding based on rational considerations. As shown in Fig. 7, the docking poses for each binder are quite similar in the native proteins and the QTY variants. Additional hydrogen bonds were observed at the head of TM helices due to the increased availability of polar sites. Hydrophilic interactions between binders and extracellular loops of GPR37 may form or disappear depending on conformational changes induced by either the design or the docking. However, one noteworthy consideration is that the pLDTT value of loop regions for AlphaFold2 predicted structures are generally low, suggesting their intrinsically disordered and flexible nature with higher energy states [69]. Thus, it is plausible that these regions may deform to accommodate for stronger interactions during the binding events. We then recomputed the complexes of GPR37 against Saposin C and Osteocalcin using AlphaFold_multimer, removed the respective binding partners, and redocked them back to the extracellular regions of the receptor using ZDOCK. The models of native and QTY GPR37 against Saposin C still exhibited aberrant N-terminal loops with slightly different docking poses and hydrogen bond interactions (Additional file 1: Fig. S3A). Yet the models of native and QTY GPR37 against Osteocalcin showed closely-matching docking poses and hydrogen bond interactions (Additional file 1: Fig. S3B). In general, similar molecular dockings between native and QTY proteins were observed in these simulations.

### Molecular docking analysis of GPR35 versus pamoic acid

The docking poses of GPR35 versus pamoic acid in native and QTY variant proteins were notably different, associated with the largest binding energy change amongst all computed pairs. To further explain this phenomenon, we carried out MD simulations on both complexes using GROMACS and Charmm36 force field [70, 71]. The simulations were conducted for 50 ns to allow the full stabilization of both binding partners in complexes (Additional file 1: Fig. S4).

**Fig. 8:**
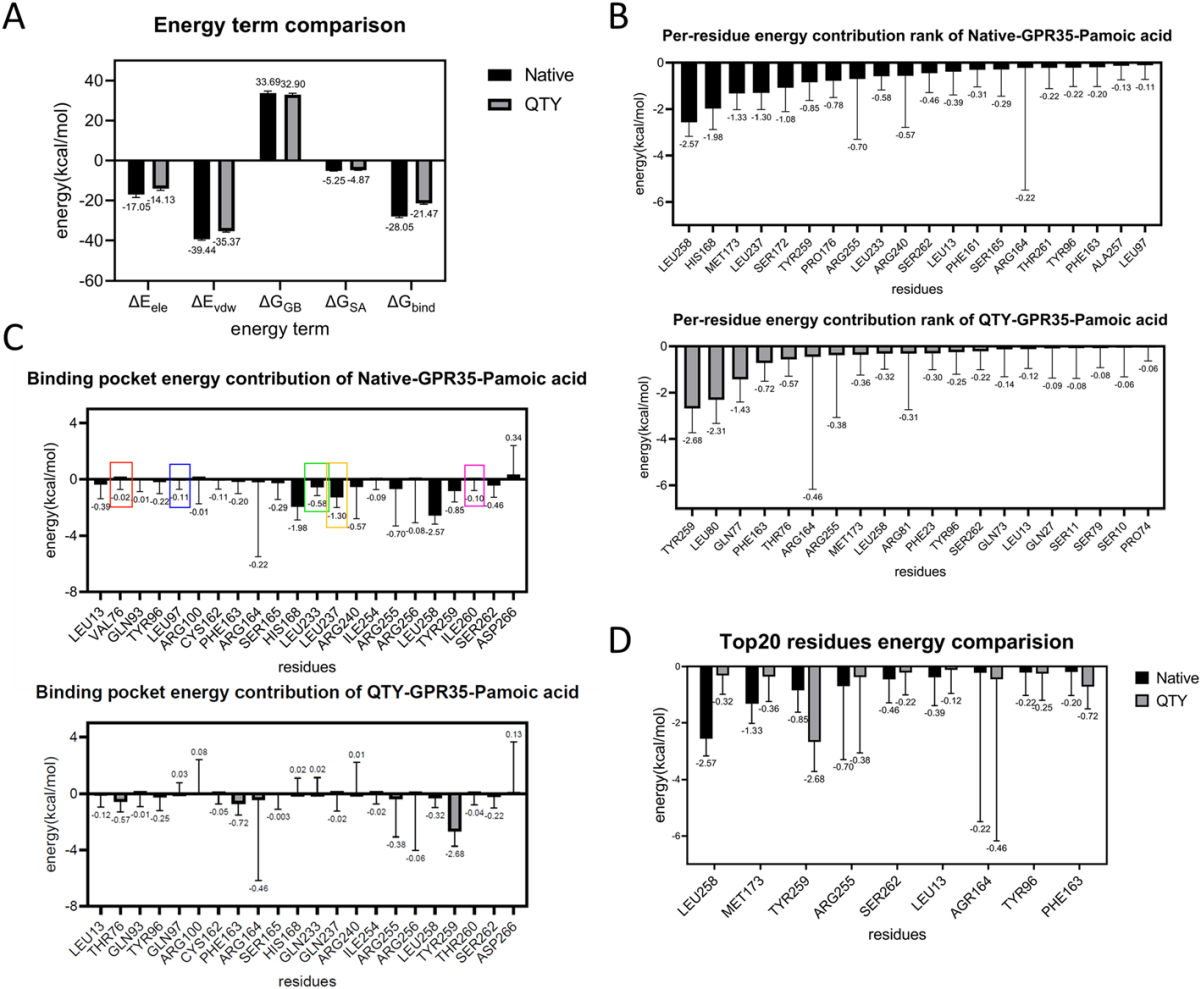
MD simulations of native and QTY variant GPR35:pamoic acid binding pairs using GROMACS. The binding free energies are calculated by MMGBSA. **A** The comparison of binding energy terms in native and QTY proteins. ΔG_bind_: free energy of binding, ΔE_ele_: electrostatic interaction energy, ΔE_vdw_: non-bonded van der Waals interaction energy, ΔG_GB_: polar solvation free energy, ΔG_SA_: nonpolar solvation free energy. **B** Top 20 residues contributing to the binding complexes. **C** Contributions from residues in the binding pocket areas to the complexes. **D** Comparison of key unchanged residues contributing to the binding complexes of native and QTY variants of GPR35 versus pamoic acid

The MMGBSA approximation was employed to calculate the binding free energies for stabilized complex structures [72]. As shown in Fig. 8A, the major energy terms that differed were ΔE_ele_ and ΔE_vdw_, representing the electrostatic interaction energy and the non-bonded van der Waals interaction energy, respectively. The decreased contributions from both terms in the QTY protein may be attributed to the inverted docking poses and more complex hydrogen bond network at the interface. These two factors combined led to a decreased binding energy between the two [36].

The hypothesis was supported by the per-residue energy contribution graph shown in Fig. 8B. Despite a few stronger interaction sites (Tyr259, Leu80, Gln77), less residues contributed moderately in the QTY variant compared to the native protein, which cumulatively led to a weaker interaction. The energy contributions from residues in the binding pockets (Fig. 8C) again agreed with the above statement where decreases in hydrophobic residue contributions (Leu13, Phe163, Leu233, Leu237, Leu258) were likely to be resulted from the outward-facing nonpolar region of the ligand in the QTY complex. Colored boxes denoted energy contributions from sites subjected to QTY substitutions. Figure 8D summarizes the top unmodified interaction sites from native and QTY proteins. It was shown that the altered binding pose significantly changed interaction sites in complexes, whereas the exclusion of the hydrophobic side of ligands from the interior of TM helices due to the additional internal hydrogen bond network likely played a critical role in this process. The observation for GPR35:pamoic acid binding pair suggested that, despite most QTY variants exhibiting high structural similarity with their native protein counterparts, the sequence change can still pose a notable impact on their interactions with certain binding partners, and should be taken into consideration for task-specific designs.

## Discussion

Transmembrane proteins are the input/output machinery of living organisms and perform an extensive variety of functions crucial to biological and pathological processes, including mechanistic pathways essential for the progression of various types of cancers [73]. They bear great importance in understanding tumor pathogeneses with implications for cancer treatments and patient prognosis [9–12, 74]. Many types of membrane proteins also contain well-defined binding pockets that may be directly adopted as targets for therapeutics and modern medicine [1, 75, 76].

Yet to date, the systematic correlation between membrane protein types and diseases is still only at the genetic level, where gene profiling techniques were used to reveal overexpressed species in certain cancers [16, 17]. Understanding of molecular mechanisms and functional roles in association with specific pathogenesis is still lacking [4], primarily due to the inherent hydrophobicity, the difficulty to express in native conformations, and the instability ex vivo [23, 77]. The deep-learning based AlphaFold2 partially resolved the issue by providing highly accurate structure predictions for these hard-to-work-with protein species. Yet computed structures still need to be experimentally verified with subsequent mechanistic studies at the molecular level [31].

By establishing a dedicated cancer-related membrane protein database, our work contributes to the current status quo of research in two aspects. Firstly, the machine-learning based correlation between protein functions, classifications and cancer types encode essential molecular information contributing to the key mechanistic pathways in tumor progressions. By reducing the high-dimension matrix with all critical functional descriptions into 3-dimensions, the spatial distribution of datapoints may be used to predict previously inapparent relations between adjacent proteins involved in mechanistically connected pathways for specific pathogenesis that are not directly revealed by genetic level analysis.

On the other hand, to circumvent the difficulties in membrane protein study induced by hydrophobicity, we have used a rational design tool called the QTY code, which regulates protein solubility through pairwise amino acid substitutions [35]. The methodology was experimentally demonstrated on 12 types of membrane receptors including 7TM GPCRs [32, 35, 36, 45], with more types computationally designed and reported [32, 37, 38]. It was also adopted to identify essential structural domains for ligand binding and proteins’ regulatory roles in vivo [35, 36]. The water-soluble variants can greatly benefit the molecular understanding on native proteins by providing physical simulators of the latter, due to their structural and functional similarities. However, no crystal structure or ligand docking studies have been conducted to date, both of which would further demonstrate the QTY code’s applicability to facilitate membrane protein research.

We partially solved the problem by conducting ligand docking and molecular simulations in the current work. With 5 selected membrane proteins that differ in TM helices, classifications, and functions, we compared the bindings between native and QTY variants against known ligands. While all 5 examples exhibited high similarities in protein characteristics, AlphaFold2 predicted structures, PrankWeb predicted binding pockets, and slightly varied docking poses, some complexes showed notable changes in both ligand orientation and binding energies, including 2/3 complexes with MGAT3 and 1 complex with GPR35. By MD simulation, we found that the reduced hydrophobic interactions between ligand and QTY protein are accountable for the differences. It appeared that TM enzymes were most susceptible to such changes which might negate their catalytic functions. Receptors were slightly affected, while the structure and functions of channel-forming proteins were best retained with the QTY design. Our observations further suggested the applicability of QTY code on different classes of proteins where task-specific designs need to be taken into consideration for species more susceptible to the formation of internal hydrogen bond networks.

The work presented in this manuscript provides a bioinformatic guideline to determine whether or not a specific QTY design on a membrane protein should be adopted for experimental studies or applications. Superimpositions between the native and QTY variant proteins, as well as the corresponding RMSD values are the primary factors to be considered. Designs with RMSD ≤ 2 Å are generally considered conformationally similar to their native counterparts and suitable for subsequent uses. Higher H_Ƴ_ change with nearly vertical lines (little R_H_ change, Fig. 4F) indicates superior design efficiency in enhancing protein solubility without changing its secondary structure, which in combination are positive selection factors. Prankweb predicted binding pocket is another factor to be considered but not necessarily determined upon whether a design should be pursued. The docking pose evaluations are typically conducted by end-users to evaluate the feasibility of ligand-specific applications.

However, there are still a few limitations in the current study that can be worked on to further improve our database and designs. Firstly, the extraction of keywords was processed with the classic TF-IDF algorithm, which was effective in completing the task but fell short in context analysis and lacked biological specificity. We plan to evaluate new language models on this task with extensive training on biology texts to optimize the representation of protein functions. In addition, to further validate the predicted function-based protein-cancer relations in our database, a large language model-based algorithm can be built to conduct literature-wide search and validation. On the other hand, the QTY designs in our database were conducted using the “simple design module” on the PSS server, which featured high efficiency but lacked customization for each protein. In combination with the above-mentioned large language model, we plan to further optimize the QTY design process for individual membrane protein optimization that best retain their functions in specific pathogenesis. MD simulations and resolving the crystal structures of QTY variant proteins beyond AlphaFold2 models will also further benefit both the understanding of these designs and their uses as physical simulators of the native proteins.

In summary, our database provides well-documented information about molecular information of membrane proteins and its expressions in cancers. It pushes beyond the genetic level analysis to reveal undiscovered connections between proteins’ molecular functions and pathogenesis by machine-learning enabled predictions. QTY-code enabled water-soluble designs of membrane proteins are presented as an additional solution for the lack of information on membrane proteins. The variants can be experimentally adopted to facilitate ligand identification from a biophysiochemical aspect and mechanistic pathway studies of critical native proteins. They may also potentially serve as novel targets for immunotherapy in cancer treatments. The discovery, verification and modulation of novel cancer-related molecular mechanisms can not only benefit the scientific understanding of initiation and progression of specific malignancies, but also add tools that can help to concur these diseases.

## Methods

### CrMP-Sol (Cancer-related Membrane Protein and Solubilization database)

The database is accessible at Metagene platform of Zhejianglab (https://bio-gateway.aigene.org.cn/g/CrMP). The website does require registration but is free to use.

### Data acquisition and protein classification

Functional descriptions of each protein were obtained from Uniprot (https://www.uniprot.org/) and associate with corresponding entries. The classification of proteins was based on their names, keywords, and functional descriptions on corresponding Uniprot pages. Protein entries lacking meaningful keywords and functional descriptions are assigned into the “other” category.

### Keyword extraction of protein functions

TF-IDF was conducted for keyword extraction. We first performed data cleaning and use regular expression to specify search strings in protein function descriptions. PubMed IDs and punctuation marks were removed to reduce meaningless texts during encoding. We then used the CountVectorizer function to extract text features from proteins' functional descriptions. Common English stopwords such as articles and conjunctions were also removed from the text during this process. The number of feature words can be adjusted by changing the 'max_feature' parameter in this function. Subsequently, we use the TfidfTransformer function to encode the descriptions into a [1309 × max_feature] matrix.

### Dimension reduction and visualization

The UMAP algorithm was used to perform the dimension reduction on encoding matrix above. The parameters are set as follows: n_neighbors = 10, n_components = 3, min_dist = 0.5, metric = 'correlation', random_state = 16. A [1309 × 3] matrix was obtained as the final output. Protein classifications and related cancer types are added as labels to the above matrix. The interactive visualization in the 3D coordinate system was achieved using three.js (https://threejs.org/).

### QTY code design

QTY code design on all 1309 membrane proteins were conducted using a server we have previously established (https://pss.sjtu.edu.cn/) [44]. FASTA sequences of each entry in the dataset was obtained from Uniprot using a custom Python code. The sequences were then converted into their soluble versions following the principles outlined by QTY method, namely all hydrophobic L, I and V, F were pairwisely substituted by Q, T, and Y in denoted TM domains. The information regarding starts and ends of each TM helices were extracted from the topological domain section in Uniprot database. Automated design was then conducted using the “simple design module” on the server.

### Sequence alignment and property calculation

The native protein sequences for cancer-related membrane proteins and their QTY-variants are aligned using the same methods as described previously [32, 38]. The website ExPASy (https://web.expasy.org/protparam/) was used to calculate the MW and pI values of the proteins.

### Structure prediction and superimposition

AlphaFold2 was used to predict structures for all cancer related membrane proteins in QTY forms, the service of which is freely provided by Zhejiang Gene Computation Platform (https://cloud.aigene.org.cn/). The predicted structures for native proteins were directly obtained from Uniprot as provided by the European Bioinformatics Institute (https://alphafold.ebi.ac.uk). Structure files for 5 selected proteins were then downloaded and superimposed using PyMOL with RMSD calculated. A Python script was programmed to calculate the RMSD values in batch with PyMOL 2.4.1.

The secondary structure of proteins was predicted using DSSP software [78], and the percentage of helical content changes was normalized to a polar coordinate system to the 180° scale. Proteins with pI > 7 were placed above the horizontal line and those with pI < 7 were placed below the horizontal line. Datapoints were color-coded by protein MW weight and placed according to respective RMSD changes between the two protein variants.

### Hydrophobicity prediction

The surface hydrophobic patch was visualized using a script developed by Hagemans et al*.* for highlighting with the YRB scheme [79]. The standalone software ProPAS was used for the prediction of the protein features including pI, MW, and hydrophobicity [80]. The T_m_ value was calculated using T_m_ Predictor localized software with the default T_m_ reference matrix [63].

### Ligand docking comparison

The PrankWeb server (https://prankweb.cz/) was used to predict the binding pockets of native and QTY versions of 5 exemplary proteins based on their AlphaFold2 predicted structure models. Predictions were ranked based on their scores and selected from the top 3 candidates for docking analysis on a rational basis.

The structures for micromolecular ligands were downloaded from PubChem website (https://pubchem.ncbi.nlm.nih.gov/) and converted into.pdb file using OpenBabel. GCDC was extracted from a complex structure from PDB entry: 7ZYI. After preprocessing of the ligand and protein (add polar hydrogen atoms and torsion), the dockings processes were performed by AutoDock Vina with PrankWeb predicted pocket center and defined box dimensions between 15 AND 25 Å.

Dockings were performed for at least 3 times for each protein–ligand pair. The top-ranking conformations appeared 3 times were selected for presentation. The results were then visualized by PyMOL. Native proteins are colored green, and QTY proteins are colored cyan. The ligands are shown in yellow, and the hydrogen bonds are shown in magenta. Residues having polar contact with ligands are shown as stick, with labels displayed. All atoms in proteins are added with polar hydrogen atoms.

The docking between GPR37 and protein binders were performed by Linux ZDOCK 3.0.2. The structure of Saposin C is obtained from PDB (PDB ID: 2GTG) while those for Parkin (Uniprot ID: O60260) and Oseocalcin (Uniprot ID: P02818) were obtained by AlphaFold2 prediction. The large N-terminus 1–255 residues with very low pLDTT (< 50) were removed before docking. The intracellular loops and C-terminus of the proteins were blocked from docking simulations. The dockings processes were conducted at 6°rotational sampling density for maximal precision. Top 100 complexes with highest scores were selected out of 54,000 generated poses. Docking complexes within top 3 were inspected and selected for presentation. The docking results were visualized by PyMOL. Native proteins are colored green, and QTY protein are colored cyan. The ligands are shown in yellow, and the hydrogen bonds are shown in magenta. Residues having polar contact with ligands are shown as stick, with labels displayed. All atoms in proteins are added with polar hydrogen atoms.

### MD simulation

MD simulations of native and QTY variant GPR35 versus pamoic acid complexes were performed using GROMACS v2022.3 with the Charmm36 force field. The topology files in the Charmm force field of protein were generated by GROMACS, and the topology files in the Charmm force field of ligand were generated by CGenFF website (https://cgenff.umaryland.edu/). The complexes were immersed in the periodic orthorhombic water box (TIP3P) with added appropriate number of Cl^−^ ions to neutralize the systems. The Steepest Descent (SD) algorithm was used to perform energy minimization. The system was equilibrated by two steps: a 100 ps NVT process at 310 K, and a 100 ps NPT process at 1 bar with position restraints (1000 kJ/mol) on the heavy atoms of the protein and ligand. Subsequently, 50 ns MD was performed at 300 K with trajectory saved every 50 ps. After the backbone of proteins stabilized, the binding free energies were calculated using MMGBSA with the following equation:

$$\Delta {\text{G}}_{{{\text{bind}}}} = {\text{G}}_{{{\text{complex}}}} - {\text{G}}_{{{\text{ligand}}}} - {\text{G}}_{{{\text{receptor}}}} = \Delta {\text{H}} - {\text{T}}\Delta {\text{S}}$$

Where

$$\begin{aligned} & \Delta {\text{H}} = \Delta {\text{E}}_{{{\text{MM}}}} + \Delta {\text{G}}_{{{\text{polar}}}} + \Delta {\text{G}}_{{{\text{nonplar}}}} \\ & \Delta {\text{E}}_{{{\text{MM}}}} = \Delta {\text{E}}_{{{\text{bond}}}} + \Delta {\text{E}}_{{{\text{angle}}}} + \Delta {\text{E}}_{{{\text{dihedral}}}} + \Delta {\text{E}}_{{{\text{ele}}}} + \Delta {\text{E}}_{{{\text{vdW}}}} \\ & \Delta {\text{G}}_{{{\text{polar}}}} = \Delta {\text{G}}_{{{\text{GB}}}} \\ & \Delta {\text{G}}_{{{\text{nonplar}}}} = \Delta {\text{G}}_{{{\text{SA}}}} \\ \end{aligned}$$

where ∆E_MM_: electrostatic interaction energy; ∆E_ele_: gas-phase molecular mechanics energy; ∆E_vdW_: non-bonded van der Waals interaction energy; ∆G_polar_: polar solvation free energy; ∆G_nonpolar_: nonpolar solvation free energy; ∆G_polar_ and ∆G_nonplar_ were calculated by Generalized Born Surface Area.

The 15–50 ns trajectory of native GPR35:pamoic acid and 10–50 ns trajectory of QTY GPR35:pamoic acid were extracted per 1 ns to generate frames for binding energy calculations. All residues were calculated to provide a ranking of respective contributions. For calculation in binding pockets, residues in overlapping sites of native and QTY variant proteins within 6 Å were presented for comparison. Mutated residues were marked with boxes in different colors.

### Supplementary Information

**Additional file 1**. Supplementary Materials.

## Data Availability

All data supporting this study and its findings are available within the article, in associated files, and accessible at Metagene platform of Zhejianglab (https://bio-gateway.aigene.org.cn/g/CrMP).

## Declarations

### Accessibility

The database is accessible at Metagene platform of Zhejianglab (https://bio-gateway.aigene.org.cn/g/CrMP). The website does require registration but is free to use.
