# Supplementary material for: CrMP-Sol database: classification, bioinformatic analyses and comparison of cancer-related membrane proteins and their water-soluble variant designs
Source: BMC Bioinformatics. 2023 Sep 25;24:360. doi: 10.1186/s12859-023-05477-9 (PMC10518928; doi:10.1186/s12859-023-05477-9)
Supplement: Supplementary file 1 — Additional file 1. Supplementary Materials. [file 12859_2023_5477_MOESM1_ESM.pdf]

Supplementary Materials for

CrMP-Sol database: classification, bioinformatic analyses and comparison of cancer-related membrane proteins and their water-soluble variant designs

Lina Ma[1#], Sitao Zhang[1#], Qi Liang[2], Wenting Huang[1], Hui Wang[1], Emily Pan[4], Ping Xu[1], Shuguang Zhang[3], Fei Tao*[1], Jin Tang*[2], Rui Qing*[1]

[#]: These authors contributed equally to this work.
*: To whom correspondence should be addressed.

Email: ruiqing.br@sjtu.edu.cn, taofei@sjtu.edu.cn, jin.tang@zhejianglab.com

This PDF file includes:

Figures. S1 to S4

Tables S1

Figures

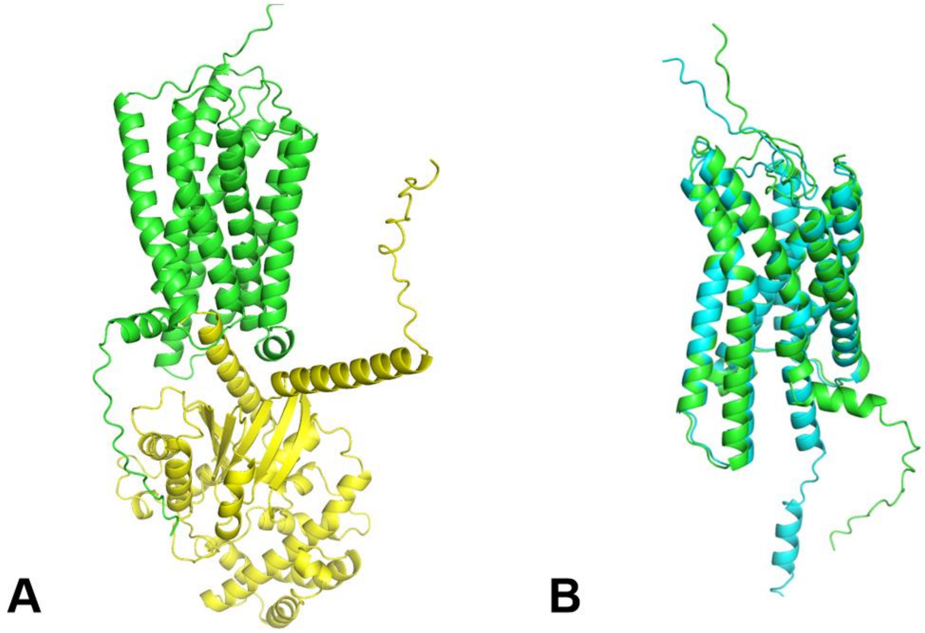

Fig. S1 (A) The GPR35+G_α complex modelled by AlphaFold_multimer. GPR35 is colored green, and G_α is colored yellow. (B) The superimposition between GPR35 modelled by AlphaFold2 and AlphaFold_multimer. GPR35 modelled by AlphaFold_multimer is colored green, and GPR35 modelled by AlphaFold2 is colored cyan.

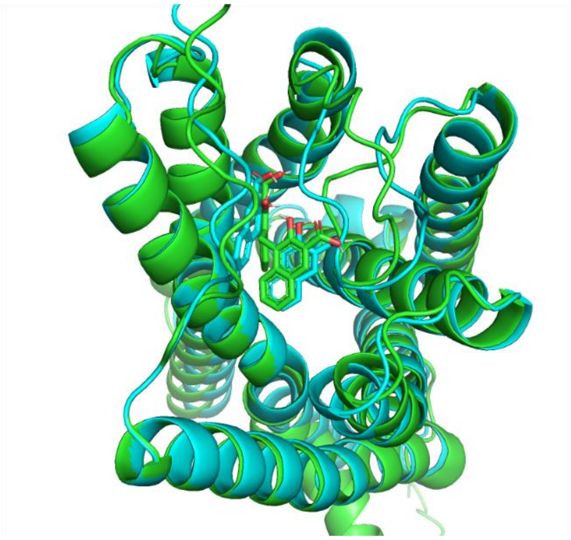

Fig. S2 The comparison of Pamoic acid-docking pose with AlphaFold_multimer-modelled GPR35 complex (green) and AlphaFold-modelled GPR35 (cyan).

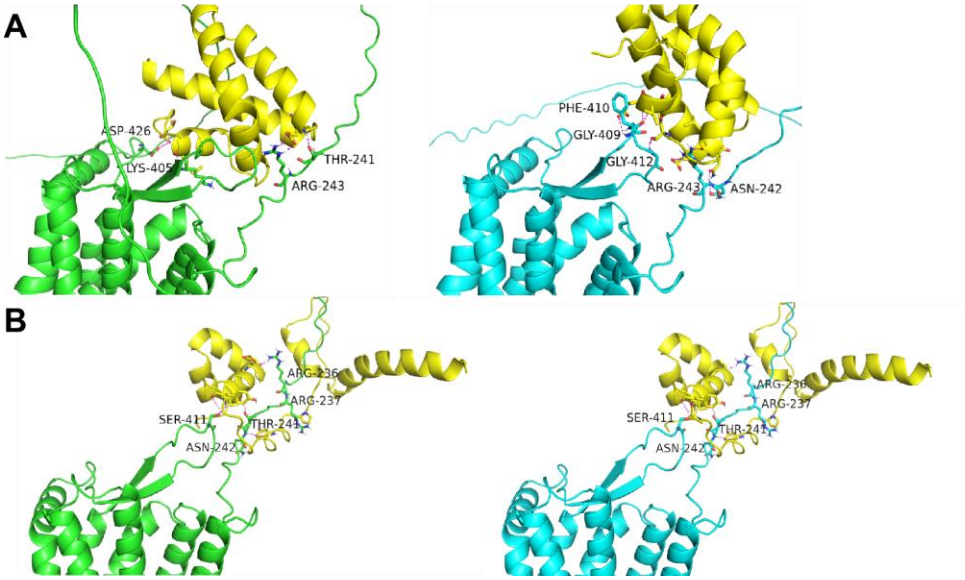

Fig. S3 The docking results of native (green) and QTY (cyan) variants of GPR37 against (A)Saposin C and (B) Osteocalcin using AlphaFold_multimer models. The ligands are colored yellow. The residues of native and QTY GPR37 variants involved in hydrogen bonding interactions are shown in stick and labeled by residues name. The hydrogen bonds are colored by magenta.

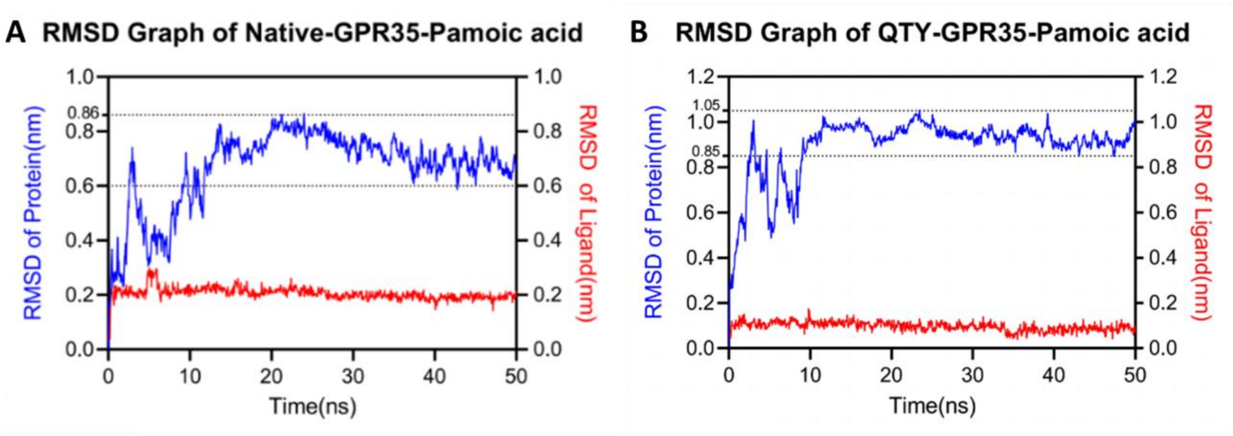

Fig. S4 RMSD change from initial complex models for (A) native and (B) QTY GPR35-pamoic acid complex.

Tables

Table S1 Z-score values of AlphaFold2 predicted structures for native and QTY variant proteins

Uniprot ID	Protein Name	Z-score
Q9HC97	Native GPR35	-5.51
	QTY GPR35	-4.81
O15354	Native GPR37	-3.69
	QTY GPR37	-3.05
Q14973	Native SLC10A1	-4.87
	QTY SLC10A1	-2.69
Q9UHC9	Native NPC1L1	-12.62
	QTY NPC1L1	-11.23
Q86VF5	Native MOGAT3	-5.58
	QTY MOGAT3	-6.46
